# Effects of Ultrasound-Guided Lower Extremity Nerve Blocks for Below-Knee Procedures in the Emergency Department of a Tertiary Care Hospital, Central Gujarat

**DOI:** 10.7759/cureus.41450

**Published:** 2023-07-06

**Authors:** Shreyas K Patel, Rina Parikh, Himanshu Gupta, Krunalkumar Pancholi, A. K Saxena, Bansari Chawada, Kalpita S Shringarpure, Kedar Mehta, Parag Chavda

**Affiliations:** 1 Department of Emergency Medicine, Parul Institute of Medical Science and Research, Vadodara, IND; 2 Department of Emergency Medicine, Sir Sayajirao General (SSG) Hospital, Medical College Baroda, Vadodara, IND; 3 Department of Emergency Medicine, Jaipur National University (JNU) Institute for Medical Sciences and Research Centre (IMSRC), Jaipur, IND; 4 Department of Emergency Medicine, Parul Institute of Medical Sciences and Research, Vadodara, IND; 5 Department of Community Medicine, Sir Sayajirao General (SSG) Hospital, Medical College Baroda, Vadodara, IND; 6 Department of Preventive and Social Medicine and Public Health, Sir Sayajirao General (SSG) Hospital, Medical College Baroda, Vadodara, IND; 7 Department of Community Medicine, Gujarat Medical Education and Research Society (GMERS) Medical College - Gotri, Vadodara, IND; 8 Department of Preventive Medicine, Gujarat Medical Education and Research Society (GMERS) Medical College - Gotri, Vadodara, IND

**Keywords:** usg-guided procedures, usg-guided nerve block, popliteal nerve block, femoral nerve block, india, below knee procedures, nerve blocks, ultrasound

## Abstract

Background and objectives

Ultrasound-guided femoral and popliteal sciatic nerve blocks are useful adjuncts for many below-knee procedures like debridement, amputation, etc. The objectives of the study were to find the efficacy and feasibility of the ultrasound-guided combined femoral and popliteal sciatic nerve block for below-knee procedures in the Emergency Medicine Department (ED).

Methodology

This prospective clinical study was carried out over three months in ED. A total of 30 patients undergoing below-knee procedures were included in the study. Femoral and popliteal sciatic nerve blocks were administered to each patient using the high-frequency linear ultrasound probe by emergency physicians trained in ultrasound. The effect of blocks, amount of local anesthetic (LA) used, duration of the procedure, and post-block analgesia were recorded. Patients were monitored for possible complications, if any. Data were entered and analyzed using a Microsoft Excel worksheet.

Results

The average volume of LAs required was around 34.5 cc for both blocks combined. No complications like vascular puncture or nerve injury were reported during the study. The time taken to complete the procedure was around 33 minutes, and the average time to achieve sensory block was around 9 minutes after completing the procedure.

Conclusions

An ultrasound-guided combined femoral and popliteal sciatic nerve block is an effective and feasible procedure and thus should be considered in ED for below-knee procedures.

## Introduction

General or spinal anesthesia in lower extremity procedures among advanced-age patients carries an increased risk of mortality and various complications such as pain, respiratory problems, headache, hypotension, and urinary retention [1]. Effective and prolonged anesthesia for lower limb procedures can be achieved with regional lower limb blocks [1].

The ideal in the practice of regional anesthesia would be the ability to deliver the right dose of local anesthetic precisely to the target nerve without incurring any damage to the nerve or its related structures. This is commonly achieved by using needles and catheters, guided mostly by knowledge of anatomy supplemented by electrical nerve stimulation or the elicitation of paraesthesia in resource-limited setups like government hospitals in India. Unfortunately, this is a blind process; however, modern imaging techniques can help to overcome this limitation. Ultrasound-aided nerve blocks have been reported in the anesthetic literature since 1978, with an increase in interest from the mid-1990s, probably as a result of improvements in ultrasound equipment [2].

Ultrasound-guided femoral and sciatic nerve blocks are useful adjuncts for postoperative analgesia and managing post-amputation limb pain [3]. It has several beneﬁts, including faster onset and reduction in the dose of local anesthetic as compared to blind nerve blocks for below-knee procedures in the Emergency Department (ED) also [4]. Ultrasound imaging techniques enable the emergency physician in the ED to secure an accurate needle position and monitor the distribution of the local anesthetic solution in real time. Modern ultrasound equipment is cheaper and more portable and produces better-quality imaging [5].

The studies reporting the efficacy of femoral and popliteal sciatic nerve blocks individually are available from India. To achieve analgesia of the whole limb simultaneous block at both the nerves is required. However, there is no study reporting the usage of combined ultrasound-guided femoral and sciatic nerve blocks for below-knee procedures in the ED. Thus, this study was conducted to examine the efficacy and feasibility of this new approach of ultrasound-guided combined femoral and popliteal sciatic lower extremity nerve blocks for below-knee procedures in the ED.

## Materials and methods

The study was conducted by emergency physicians trained in ultrasound in the ED of a Tertiary Care Hospital in Central Gujarat, India. This was an interventional study of 30 patients undergoing below-knee surgical procedures. This study was conducted for three months, from June 2014 to August 2014. All the 30 adult patients attending the ED who required lower limb nerve block for below-knee procedures were selected to participate in the study. In this study, we included adult patients requiring regional anesthesia for below-knee procedures like debridement, incision and drainage, and toe amputation. We excluded patients with pregnancy, diabetic neuropathy, allergy to local anesthetic (LA), and hemorrhagic diathesis.

The procedure of nerve block

**Figure 1:**
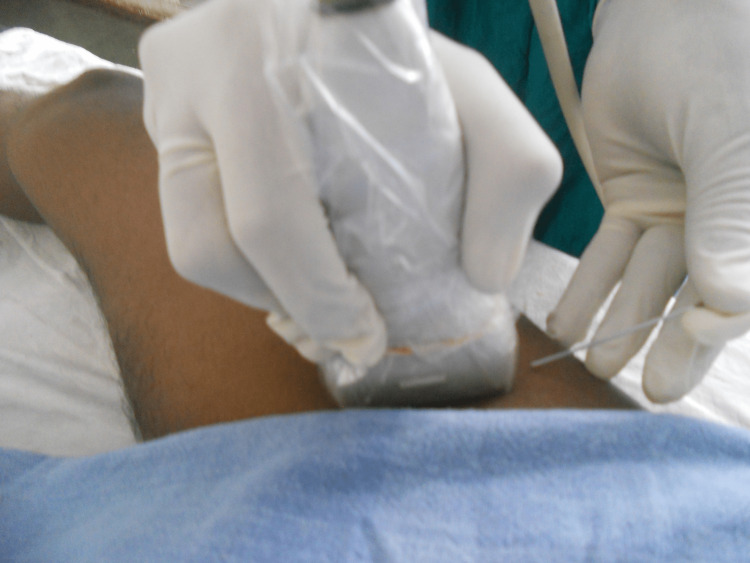
Probe position for the femoral block.

**Figure 2:**
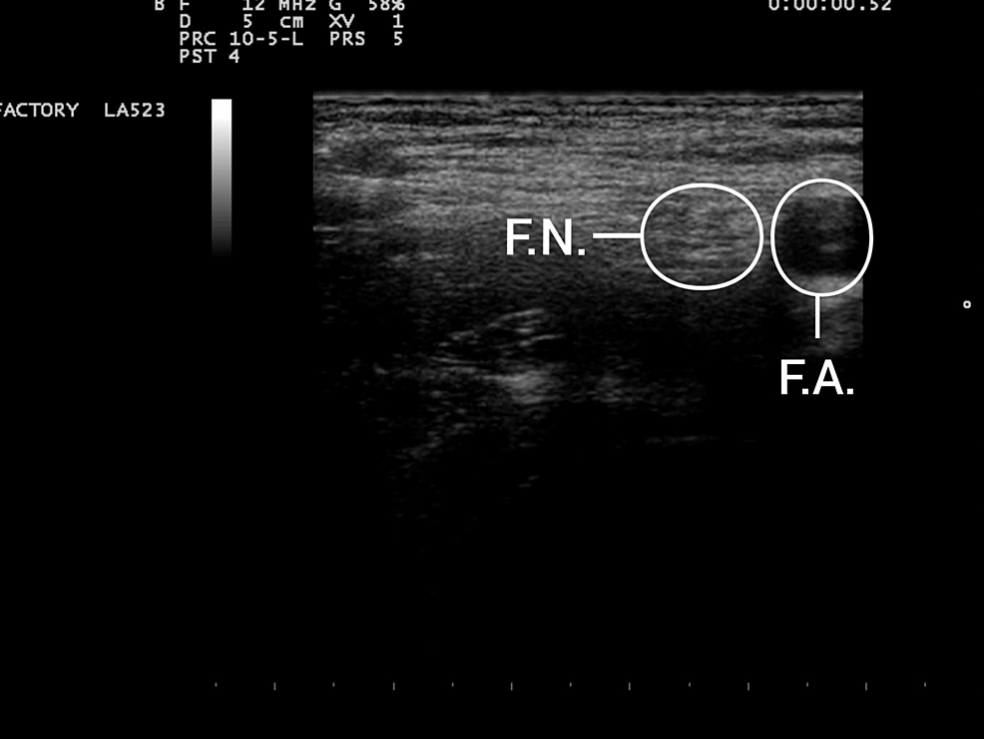
Sonographic picture of FN. FN, femoral nerve; FA, femoral artery

**Figure 3:**
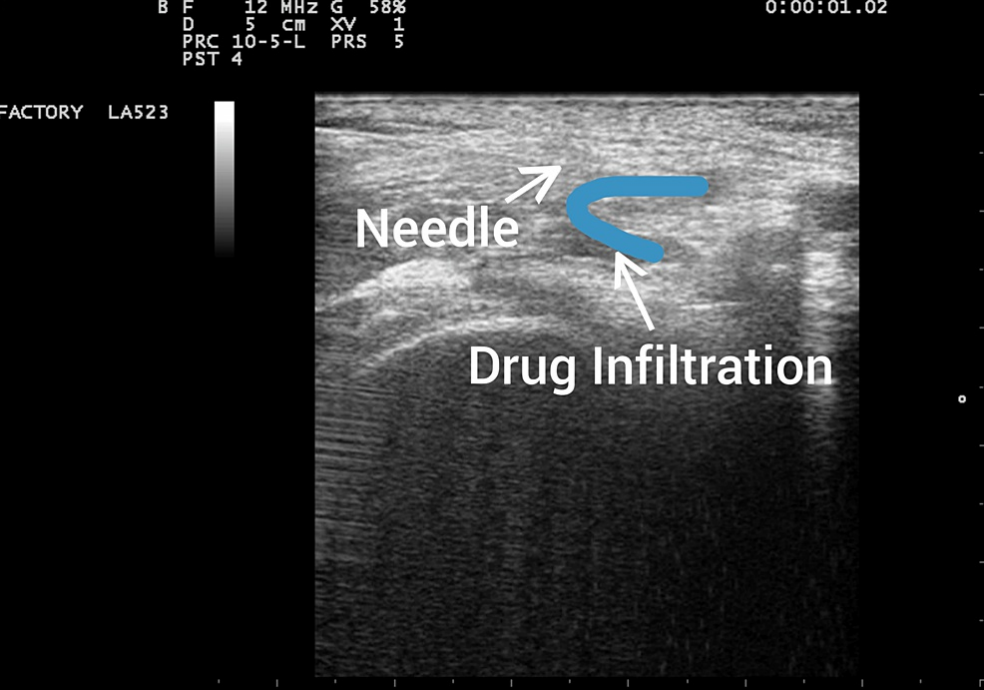
Needle insertion and local anesthetic infiltration surrounding the femoral nerve.

**Figure 4:**
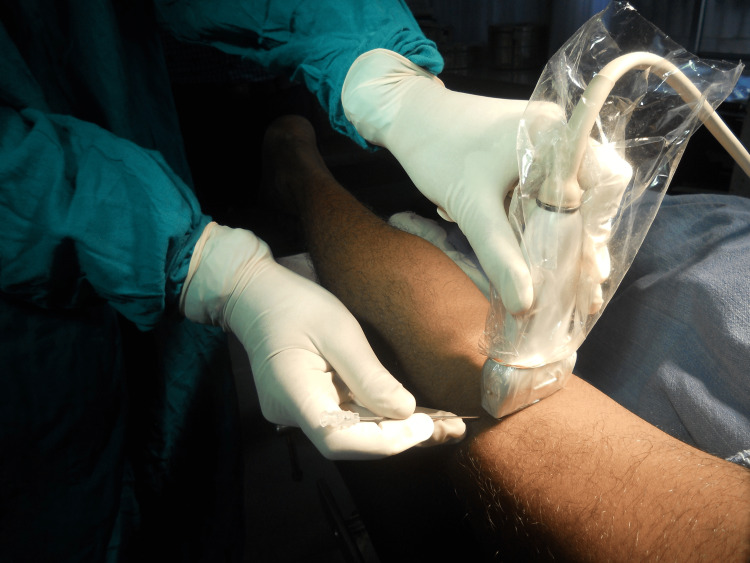
Probe position for the popliteal sciatic nerve block.

**Figure 5:**
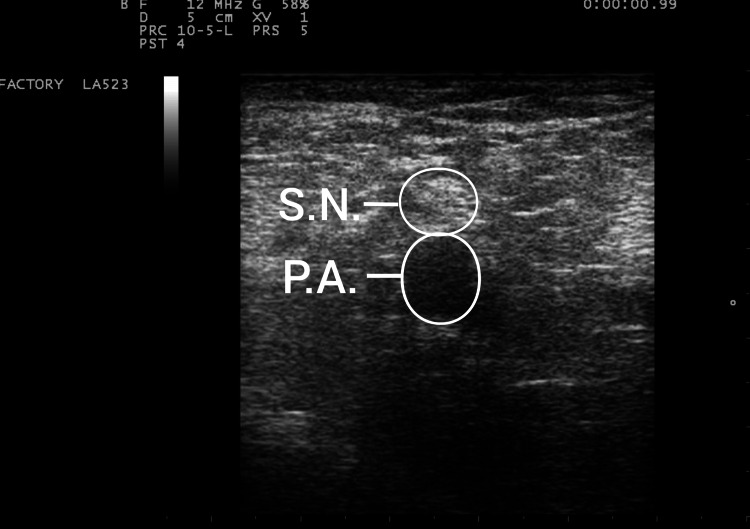
Sonographic picture of the popliteal sciatic nerve. SN, sciatic nerve; PA, popliteal artery

**Figure 6:**
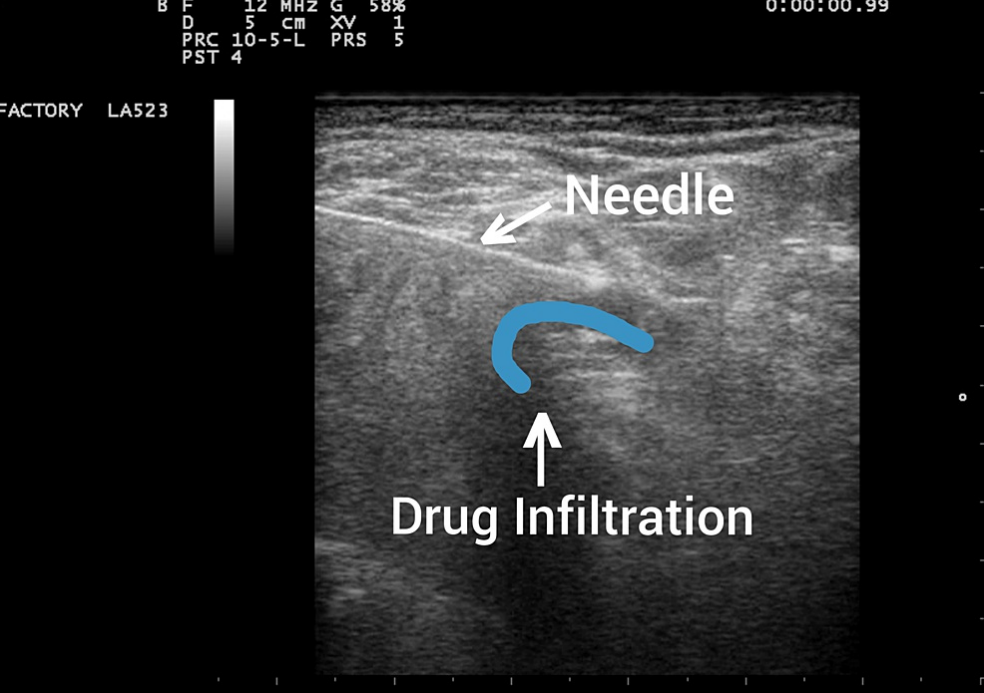
Needle insertion and local anesthetic infiltration surrounding the popliteal sciatic nerve.

LA mixture was prepared using 2% lignocaine with adrenaline 5 mg/kg of lean body weight + 0.5% bupivacaine 1 mg/kg of body weight + 8.4% sodium bicarbonate 1 mL per 10 mL of LA. The total volume of 50 mL is achieved by adding sterile water. An ultrasonography machine (Esaote MyLab30, Esaote India (NS) Ltd., Ahmedabad, Gujarat) with a linear transducer (8-14 MHz) was used. The sterile technique was performed with the help of a sterile sleeve and gel, standard nerve block tray (i.e., sterile towel and gauze piece, 10 mL syringes for LAs, 5 ml syringe, and a 24-gauze needle for skin inﬁltration, 100-mm-long 18G Quincke spinal needle for infiltration of an LA drug, sterile gloves, and antiseptic solutions). After taking informed and written consent, the patient was given an injection of midazolam 1-2 mg intravenously (IV) 20 minutes before starting the procedure. The transducer probe was covered with a sterile sleeve. After antiseptic painting and draping, the transducer was placed in an appropriate position. Standard noninvasive monitors were applied, and all emergency drugs were kept available. Ultrasound-guided nerve block procedure is illustrated in Table 1 and Figures 1-6.

**Table 1:** Procedure for nerve block. LA, local anesthetic

	Femoral nerve block	Popliteal sciatic nerve block
Patient’s position	Supine	Prone
Transducer position	Parallel to the femoral crease to identify femoral vessels and femoral nerve	Parallel to the popliteal crease, the transducer may need to be moved cephalad to identify the popliteal vessels and the sciatic nerve.
Nerve was identiﬁed as	Hyperechoic, roughly triangular or oval structure, lateral to femoral vessels, and underneath the fascia iliaca	Hyperechoic, oval, or round structure with a stippled or honeycomb pattern, superior and lateral to popliteal vessels
Initial depth setting (highly dependent on patient size)	4 cm	5 cm
Local inﬁltration was done at the presumed needle puncture site.		
LA required (till the nerve was surrounded by drugs)	15-20 mL	20-30 mL
Once the needle tip was adjacent to the nerve, after hemonegative aspiration, 1-2 mL of LA was injected and its spread around the nerve was conﬁrmed and then additional LA was injected.		
Needle Insertion	In plane, lateral to medial	In plane, lateral to medial
Ideal spread of LA	Beneath the fascia iliaca around the femoral nerve	Around the sciatic nerve

Sensory assessment using the pinprick method was performed every ﬁve minutes after completion of popliteal sciatic nerve block following the femoral nerve block procedure until the complete sensory loss was achieved (i.e., loss of sensation over medial and lateral malleoli). The patient was observed for six hours and was assessed every hour for a visual analog scale (VAS) score. When the VAS score was >3, analgesia was given in the form of oral analgesic tablets. Complications, if any, were noted by the emergency physician. The patient was discharged after regaining the total sensory and motor function of the lower limb.

Measurement methods

Calculation of time required for the procedure was done as follows: The initiation point for the femoral nerve block procedure was from time point *a* when the patient was placed in the supine position for this block. The endpoint of this procedure was at point *b* when the femoral nerve was found surrounded by LA on ultrasonography. Immediately after getting point *b*, the patient was shifted to a prone position for the popliteal sciatic nerve block (initiation of the popliteal sciatic nerve block procedure). When the popliteal sciatic nerve was surrounded by LA, time was noted as point *c* (completion of the popliteal sciatic nerve block procedure). The sensory assessment was done every ﬁve minutes after point *c* until complete sensory loss over medial and lateral malleoli was achieved. This point of complete sensory loss was noted as point *d*.

Data management

Data were entered and analyzed using the Microsoft Excel worksheet.

## Results

The average age of the study participants was 50 years, with half of them classified as having the American Society of Anesthesiologists (ASA) III physical status (Table 2). Debridement was the most common indication for the nerve block procedure in this study. 

**Table 2:** Clinical and demographic details of the study participants (n = 30). Results are displayed as the number of patients out of 30, and the total percentage (%) involvement is shown in brackets. ASA, American Society of Anesthesiologists; SD, standard deviation

Parameters	Value
Age (years; mean ± SD)	50 ± 18
Weight (kg; mean ± SD)	67.7± 5.9
ASA scale, *n* (%)	
I	8 (27)
II	7 (23)
III	15 (50)
Indication for the nerve block, *n* (%)	
Debridement for cellulitis	10 (33)
Incision and drainage for abscess	7 (24)
Ulcer debridement	5 (16)
Toe amputation	3 (10)
Pain relief in below-knee crush injuries	3 (10)
Superﬁcial burns debridement	2 (7)

The ultrasound-guided combined femoral and popliteal sciatic nerve block could be performed on all 30 patients enrolled in the study. The average volume of local anesthetic needed for the combined nerve block was 34.5 cc. The mean time taken to achieve the complete sensory block was 42 minutes. There were no complications recorded among any of these patients (Table 3).

**Table 3:** Nerve block characteristics during below-knee procedures among the study participants (n = 30). *Time taken from point *a* to point *b* ^#^Time taken from point *b* to point *c*. ^$^Time taken from point *c* to point *d*. ^%^The time taken from point d to the point where the patient feels pain with a visual analog score >6.

Parameters	Value (mean ± SD)
Amount of drug volume use	34.5 ± 6.86 cc
Time taken to complete the femoral nerve block^*^	13.2 ± 4.91 minutes
Time taken to complete popliteal sciatic nerve block^#^	20.05 ± 7.49 minutes
Time taken to achieve sensory blockade^$^	8.75 ± 2.82 minutes
Duration of post block analgesia^% ^	371.25 ± 31.11 minutes
Complication rate	0%

## Discussion

In our study, we found that sonography-guided femoral and popliteal sciatic nerve block is a safe and easy procedure that can be learned and performed with minimal sonographic training by emergency physicians. Identiﬁcation of both the femoral and sciatic nerves under sonographic guidance was enhanced by surrounding vascular, muscular, and fascial structures which served as good sonographic landmarks. This allowed the ultrasound-guided puncture to be more precise thus minimizing the risk of accidental vascular puncture. Many studies have also concluded that sonography-guided nerve block provides the advantage of direct visualization of the nerves to be blocked compared to the blind anatomical landmark technique. This allows complete inﬁltration surrounding the nerve with LA, allowing accurate drug delivery, and thus, the number of drugs used can be reduced [6-8].

Studies performed by Marhofer et al. found that the onset time of the block was significantly shorter and quality was significantly better in the ultrasound group compared to the nerve stimulator group [6,9]. In our study also, the time taken to achieve a sensory blockade was around 9 minutes after completion of the procedure, although we did not have any comparison group. There were no complications reported in our study; it might be because of a smaller sample size of 30 patients. However, Lewis et al. found that nerve blocks when performed using ultrasound guidance were not completely risk-free but would have fewer complications such as *pins and needles* or accidental punctures of blood vessels [10].

In our study, we performed the nerve block for various below-knee surgical procedures like debridement for cellulitis, incision and drainage for abscess, ulcer debridement, toe amputation, pain relief in below-knee crush injuries, and superﬁcial burns debridement irrespective of their ASA status. We found that half of the patients were of ASA III status, which might be because of a higher risk of complication with spinal/general anesthesia, unfit for general anesthesia or to reduce the hospital stay/ burden. As we were trained in the use of ultrasonography, we could accurately identify the nerve and deliver the drug around the nerve so that the amount of drug required to achieve sensory blockade could be reduced. Various other studies also found significant reductions in the volume of anesthetic drugs required to produce a surgical block under ultrasound guidance [6-8].

The advantage of this procedure lies for patients with advanced physical status where general/spinal anesthesia cannot be given. In such cases, a sonography-guided regional nerve block is useful with substantially reduced anesthesia-related risk. A further advantage of this new procedure is that it does not require any extra time for the preliminaries/preparations. Moreover, with the shorter-lasting effect of the regional nerve block, the patients receiving anesthesia by this method can be discharged on the same day compared to the longer hospitalization required for general/spinal anesthesia. 

Certain possible issues while using this new technique are worth discussing here. The time required to complete this procedure can be variable and dependent on the operator and patient characteristics. The familiarity of the physician using nerve ultrasound would determine the time required to complete the procedure. The patient-related factors like the local anatomical variations at the site of the block can also alter the time required for the procedure. The nonavailability of the ultrasonography machine due to its higher cost could be the major limitation in performing the procedure. Nerve identiﬁcation based solely on ultrasound requires expertise while the use of a nerve locator along with ultrasound can be a better alternative.

It is the first study of its kind from our institution using an ultrasound-guided combined nerve block. The nerve blocks among the study participants were performed by a team of emergency physicians trained in ultrasound. Within the time duration of this study, we could enroll only 30 patients, which is a limitation of this study. 

Ultrasound-guided nerve blocks can be performed by ultrasound-trained emergency physicians and minor surgical procedures can be completed in the ED itself. Thus, the workload on the OT can be reduced and diverted for more urgent surgeries and interventions.

## Conclusions

Ultrasound-guided combined femoral and popliteal sciatic block for below-knee procedures was found effective in our study with no complications. This procedure is feasible as it can be conducted by a team of emergency physicians trained in ultrasound and facilitates an early discharge of the patient.
